# Editorial: New insights into the management of chronic idiopathic constipation: from physiopathology to novel therapeutic strategies

**DOI:** 10.3389/fphar.2026.1983671

**Published:** 2026-09-21

**Authors:** Christian Lambiase, Matteo Fornai, Maura Corsetti, Massimo Bellini

**Affiliations:** 1 Gastrointestinal Unit, Department of Translational Research and New Technologies in Medicine and Surgery, University of Pisa, Pisa, Italy; 2 Regional Center for Functional and Motility Digestive Disorders, Azienda Ospedaliero-Universitaria Pisana, Pisa, Italy; 3 Unit of Pharmacology and Pharmacovigilance, Department of Clinical and Experimental Medicine, University of Pisa, Pisa, Italy; 4 NIHR Nottingham Biomedical Research Centre (BRC), Nottingham University Hospitals NHS Trust and the University of Nottingham, Nottingham, United Kingdom; 5 Nottingham Digestive Diseases Centre, School of Medicine, University of Nottingham, Nottingham, United Kingdom

**Keywords:** chronic constipation, constipation, disorders of gut-brain interaction, fecal microbiota, laxatives

## Chronic idiopathic constipation: the challenge of heterogeneity

Chronic idiopathic constipation (CIC) is among the most prevalent and burdensome disorders of gut-brain interaction (DGBIs), yet its apparent simplicity conceals substantial biological and clinical heterogeneity (Corsetti et al., 2026).

The pathophysiology of CIC is complex and often cannot be attributed to a single mechanism.

Similar symptoms may reflect impaired colonic propulsion, altered stool hydration, visceral hypersensitivity, disturbed gut-brain communication, pelvic floor dysfunction, lifestyle factors, microbial metabolism, psychological comorbidity, or combinations of these abnormalities (Sharma et al., 2021).

This heterogeneity may contribute to variable treatment responses and persistent patient dissatisfaction despite lifestyle measures, laxatives, secretagogues, prokinetics, biofeedback and, in selected patients, procedural interventions (Harris et al., 2017; Staller et al., 2026; Lambiase et al., 2025).

The 12 contributions in this Research Topic connect evolving pathophysiological insights with practical therapeutic innovation across lifestyle and nutrition, microbial metabolism, neurobiology, pharmacotherapy, procedural optimization, complementary medicine, and safety. Collectively, they encourage a shift from symptom-driven therapy to mechanism-informed, patient-centered management of CIC.

The historical perspective offered by Corazziari provided an appropriate starting point. Innovation does not always require a new molecule. Indeed, the introduction of macrogol/polyethylene glycol (PEG) marked a major advance in CIC management, enabling effective, non-absorbed, and generally well-tolerated long-term treatment across age groups and clinical settings. This narrative review also highlighted a broader lesson: therapeutic advances are most meaningful when they combine mechanistic plausibility, safety and ease of use.

## Lifestyle and diet as biologically active treatment

Sun et al., analyzing 12,912 US adults, examined the association between the American Heart Association’s Life’s Simple 7 health metrics and chronic constipation and diarrhea. They found that greater adherence to the metrics was associated with lower odds of chronic constipation and diarrhea, supporting the view that bowel health is embedded within overall metabolic and behavioral health. Although the cross-sectional design precluded causal inference, the study placed bowel health within a broader cardiometabolic and behavioral context.

Ribichini et al. similarly Recast diet as more than bulk and water: nutrients may affect motility through enteroendocrine signaling, bile-acid metabolism, microbial metabolites, chemosensation, and meal-induced reflexes. After summarizing available evidence, they proposed the SMART (Sensory, Motor, bile Acid and Reflex Tailored) Constipation Diet, a structured framework integrating mechanistic insights to align nutrient composition, meal timing, and fermentation management with the gut-brain axis. Evidence was strongest for soluble fiber, selected fruits (e.g., kiwifruits or prunes), and some mineral waters, whereas the SMART diet remains, as acknowledged by the authors, a hypothesis-generating framework requiring validation in phenotype-based trials.

Zhang et al. provided complementary preclinical evidence. In rats with low-fiber diet-induced constipation, Tiantian Capsule (a marketed health food product formulated from several plants and fungi) improved defecation, gastrointestinal transit, and colonic histology, alongside changes in 5-hydroxytryptamine-related mediators, serotonergic and immune-associated pathways, and fecal microbiota. These convergent findings support potential interaction among diet, microbial ecology, and enteric signaling, although causality and clinical relevance remain uncertain. Standardization, component-specific studies, and controlled human trials are needed before clinical translation.

## Microbial metabolism and the gut-brain axis

Several contributions placed the intestinal ecosystem, central mechanisms and gut-brain axis at the center of therapeutic development.

Wen et al. positioned fecal microbiota transplantation (FMT) within a microbiome-bile acid-receptor axis, whereby microbial remodelling could modify bile-acid profiles and FXR/TGR5 signalling, linking microbial metabolism to secretion and motility.

Reported clinical benefits are encouraging, but FMT for CIC remains investigational because donor selection, preparation, delivery, durability, safety, and predictors of response remain insufficiently standardized.

Central mechanisms have been highlighted by two neuroimaging studies.

Zha et al. identified reduced functional connectivity between the left posterior insula and a gastric-related brain network in CIC, with weaker connectivity associated with greater symptom severity.

Xie et al. found distinct resting-state brain activity and connectivity patterns in women with CIC with versus without stress urinary incontinence, supporting shared abnormalities in bowel-pelvic floor control.

Together, these small studies support an integrated model in which interoception, motor planning, autonomic regulation, and pelvic floor comorbidity may shape the clinical expression of CIC.

Huang et al. provided a transferable phenotyping approach: among 996 patients with functional dyspepsia and/or irritable bowel syndrome, multidimensional assessment identified seven psychogastroenterological profiles. Although not specific to CIC, the study suggested how motility, systemic, psychological, and environmental dimensions might refine constipation phenotyping. Prospective validation and evidence of treatment benefit remain essential.

## Established therapy, used smartly

Two randomized trials explored constipation-directed pharmacotherapy as an adjunct to bowel preparation.

Liu et al. showed that, in 210 patients with constipation, 4 days of linaclotide combined with reduced-volume PEG achieved cleansing comparable to standard 3 L PEG, with fewer adverse symptoms, greater satisfaction and better tolerability.

Song et al. found that adding lubiprostone to PEG improved bowel cleansing quality and acceptability in patients at high risk of inadequate preparation. These studies illustrated the value of matching adjunctive treatment intensity to baseline risk rather than using uniform regimens.

## Safety and complementary approaches

Pharmacological innovation must be accompanied by careful evaluation of safety and context of use.

In the FDA Adverse Event Reporting System, Fan et al. detected disproportionate reporting signals for muscle spasms with linaclotide and plecanatide, particularly early after treatment initiation. Spontaneous reports cannot establish incidence or causality, but these signals warrant clinical awareness, vigilance and confirmatory pharmacoepidemiology.

In a network meta-analysis of 23 trials involving 2065 older adults, Wang et al. evaluated six Chinese patent medicines combined with Western therapy. Some combinations appeared beneficial, but all outcomes were supported by low- or very-low-certainty evidence, with substantial risk of bias and limited safety reporting. These results should prompt rigorous multicenter trials rather than claims of superiority.

## Toward phenotype-guided, outcome-rich care

Collectively, the articles in this Research Topic argue against a single pathway or universal solution for CIC. The collection supports phenotyping CIC across transit, evacuation, pain or hypersensitivity, pelvic-floor comorbidity, diet, medication exposure, and psychosocial context. It also supports a layered model of care that begins with safe, evidence-based measures, identifies defecatory and comorbid conditions, and integrates dietary, microbial, pharmacological, behavioural, and procedural strategies according to patient phenotype and preferences.

Treatment should address the dominant modifiable mechanism: biofeedback for defecatory dysfunction, targeted dietary intervention for suitable phenotypes, PEG as an established backbone, and secretagogues or adjuncts when symptom burden or procedural risk justifies escalation.

Outcomes should extend beyond bowel-movement frequency to straining, complete spontaneous bowel movements, abdominal symptoms, quality of life, treatment burden, durability, and patient preference.

The next-generation of research should combine mechanistic biomarkers with clinically meaningful outcomes, stratify participants by transit, evacuation, sensory, psychological, and metabolic features, and prioritize long-term effectiveness and safety. Studies should also use standardized diagnoses, distinguish CIC from IBS with constipation and defecatory disorders, and combine symptoms with objective measures when needed to clarify mechanism. Microbiome studies need functional metabolic readouts; neuroimaging requires larger longitudinal cohorts; and comparative-effectiveness trials should enrich for clinically relevant phenotypes. Pharmacovigilance signals and complementary treatments deserve the same methodological rigor as conventional drug development.

Progress in CIC will come not merely from adding therapies, but from recognizing CIC as a systems disorder and combining durable therapeutic foundations with carefully tested innovation to select and sequence them more intelligently.

The practical objective is straightforward: identify the predominant mechanism, select the least burdensome effective intervention, monitor outcomes and safety that matter to the patient, and reconsider the phenotype when response is incomplete.
